# Clinical Variables, Deep Learning and Radiomics Features Help Predict the Prognosis of Adult Anti-N-methyl-D-aspartate Receptor Encephalitis Early: A Two-Center Study in Southwest China

**DOI:** 10.3389/fimmu.2022.913703

**Published:** 2022-06-01

**Authors:** Yayun Xiang, Xiaoxuan Dong, Chun Zeng, Junhang Liu, Hanjing Liu, Xiaofei Hu, Jinzhou Feng, Silin Du, Jingjie Wang, Yongliang Han, Qi Luo, Shanxiong Chen, Yongmei Li

**Affiliations:** ^1^Department of Radiology, The First Affiliated Hospital, Chongqing Medical University, Chongqing, China; ^2^College of Computer and Information Science, Chongqing, China; ^3^Department of Neurology, Southwest Hospital, Third Military Medical University, Chongqing, China; ^4^Department of Neurology, First Affiliated Hospital of Chongqing Medical University, Chongqing, China

**Keywords:** autoimmune encephalitis, anti-N-methyl-D-aspartate receptor, deep learning, radiomics, clinical features, prognosis, predictor, multiparametric MRI (mpMRI)

## Abstract

**Objective:**

To develop a fusion model combining clinical variables, deep learning (DL), and radiomics features to predict the functional outcomes early in patients with adult anti-N-methyl-D-aspartate receptor (NMDAR) encephalitis in Southwest China.

**Methods:**

From January 2012, a two-center study of anti-NMDAR encephalitis was initiated to collect clinical and MRI data from acute patients in Southwest China. Two experienced neurologists independently assessed the patients’ prognosis at 24 moths based on the modified Rankin Scale (mRS) (good outcome defined as mRS 0–2; bad outcome defined as mRS 3-6). Risk factors influencing the prognosis of patients with acute anti-NMDAR encephalitis were investigated using clinical data. Five DL and radiomics models trained with four single or combined four MRI sequences (T1-weighted imaging, T2-weighted imaging, fluid-attenuated inversion recovery imaging and diffusion weighted imaging) and a clinical model were developed to predict the prognosis of anti-NMDAR encephalitis. A fusion model combing a clinical model and two machine learning-based models was built. The performances of the fusion model, clinical model, DL-based models and radiomics-based models were compared using the area under the receiver operating characteristic curve (AUC) and accuracy and then assessed by paired t-tests (*P* < 0.05 was considered significant).

**Results:**

The fusion model achieved the significantly greatest predictive performance in the internal test dataset with an AUC of 0.963 [95% CI: (0.874-0.999)], and also significantly exhibited an equally good performance in the external validation dataset, with an AUC of 0.927 [95% CI: (0.688-0.975)]. The radiomics_combined model (AUC: 0.889; accuracy: 0.857) provided significantly superior predictive performance than the DL_combined (AUC: 0.845; accuracy: 0.857) and clinical models (AUC: 0.840; accuracy: 0.905), whereas the clinical model showed significantly higher accuracy. Compared with all single-sequence models, the DL_combined model and the radiomics_combined model had significantly greater AUCs and accuracies.

**Conclusions:**

The fusion model combining clinical variables and machine learning-based models may have early predictive value for poor outcomes associated with anti-NMDAR encephalitis.

## Introduction

Anti-N-methyl-D-aspartate receptor (anti-NMDAR) encephalitis is the most common type of autoimmune encephalitis (AE) that targets neuronal surfaces or synaptic antigens (1). Patients present with typical neuropsychiatric syndromes, including abnormal behavior or cognitive dysfunction, speech disorders, seizures, dyskinesias, decreased consciousness and autonomic instability (2, 3). Favorable clinical outcomes critically depend on early and aggressive immunotherapy (4). First-line immunotherapies include corticosteroids, intravenous immunoglobulins (IVIg), and plasma exchange, while rituximab and cyclophosphamide are considered when the first-line treatments fail (5). Several risk factors, such as disturbance of consciousness, ICU admission and no use of immunotherapy have been demonstrated to be associated with poor prognosis in anti-NMDAR encephalitis (5–7). However, previous studies were mainly observational and retrospective, did not evaluate predictive effects, and used a variety of observation periods with mixed results (8, 9). Furthermore, several studies were conducted with AE of multiple antibody types, neglecting the differences in age distribution, clinical presentation, and prognosis across different subtypes of AE (10, 11). There is no standard tool to accurately predict long-term functional outcomes of anti-NMDAR encephalitis. Moreover, sophisticated and automated methodologies are required to improve the accuracy and efficiency of prognostic prediction.

Noninvasive MRI has been widely used for differential diagnosis and follow-up assessment in patients with anti-NMDAR encephalitis (12, 13). In contrast to traditional MRI methods, machine learning has been introduced due to its potential to reveal disease characteristics that are invisible to the naked eye (14, 15). In general, machine learning can be divided into two major categories: radiomics, where image features are manually extracted, and deep learning (DL), where computers can automatically extract content without handcrafted features but require a larger pool of training images (16, 17). Both categories have been successfully applied to provide accurate diagnosis and prognostic evaluation of neurodegenerative diseases, psychiatric diseases, and tumors (18–20). Nevertheless, to our knowledge, the application of multiparametric MRI-based machine learning for prognosis prediction in anti-NMDAR encephalitis has not been fully explored.

In this study, we aimed to conduct a two-center prospective study for the structured evaluation of clinical and machine learning features in prognosis prediction for adult patients with anti-NMDAR encephalitis. We implemented and tested the clinical model and two machine learning models (DL and radiomics) on multiparametric MRI data and compared their performance. Then, we developed a new fusion model to assess the prognosis of anti-NMDAR encephalitis, a novel machine learning framework that combines a large number of clinical variables with deep learning and radiomics features trained on multiparametric MRI through stacking algorithms. To further evaluate the performance of our new model, we used an independent external dataset for validation.

## Materials and Methods

### Study Design and Participants

Patients diagnosed with anti-NMDAR encephalitis were consecutively enrolled from two large general hospitals in Chongqing, Southwestern China between January 2012 and October 2019. Eligible patients were selected using the following inclusion criteria: (1) acute onset in patients ≥18 years old; (2) no pre-existing disability before the first clinical symptoms associated with anti-NMDAR encephalitis; (3) positive CSF and/or serum tests for NMDAR antibodies; and (4) reasonable exclusion of other diseases. The exclusion criteria were as follows: (1) a neurological disease other than anti-NMDAR encephalitis; (2) incomplete clinical information and radiological data; (3) concurrent anti-NMDAR encephalitis following a herpes simplex virus encephalitis diagnosis; (4) positive CSF and/or serum tests for another AE: a-amino-3-hydroxy-5-methyl-4-isoxazol-propionic acid receptor antibody encephalitis, contactin-associated protein 2 antibody encephalitis, leucinerich glioma-inactivated protein 1 antibody encephalitis, gamma-aminobutyric acid receptors B1/B2 receptor antibody encephalitis, voltage-gated potassium channel complex antibody encephalitis, and glutamate decarboxylase antibody encephalitis; and (5) images of poor quality or with artifacts. The flowchart of the patient selection process is presented in **Supplementary Figure 1**.

The radiographic data of anti-NMDAR patients at the acute stage in the radiology department were collected. The patients’ medical records, laboratory results and prognoses were registered and reviewed by two experienced neurologists. The standardized data collection included (1) epidemiological data such as age and gender at disease onset; (2) clinical data including typical manifestations (behaviour and cognition, memory, speech, seizures, movement disorder, loss of consciousness, autonomic dysfunction, and central hypoventilation), prodromal symptoms such as headache and fever, complications (pneumonia, hypohepatia, electrolyte disturbance, urinary tract infections and gastrointestinal bleeding), ICU admission, tracheotomy, hospitalization days, relapse, rescue, status epilepticus, physical examination results such as meningeal irritation sign and pyramid sign, time to start of treatment after symptom onset and presence of tumor; (3) laboratory results including routine CSF test parameters such as CSF cell count, glucose, chloride and protein, routine blood test parameters such as leucocyte and neutrophil and antibody titers in CSF and serum; (4) EEG, ECG and conventional MRI results; and (5) treatments including first-line immunotherapies (corticosteroids, IVIg, and plasma exchange alone or in combination), second-line immunotherapies (rituximab and cyclophosphamide alone or in combination), long-term immunotherapies (azathioprine or mycophenolate) and no use of immunotherapy.

Finally, a total of 139 patients (81 women; mean age: 33.09 ± 15.61 years) in our hospital fulfilled the eligibility criteria and were subsequently randomly divided into training (n = 97) and internal testing (42) sets at a ratio of 7:3 (see **Table 1**). To validate the clinical, radiomics and DL models, we collected clinical and brain MRI data performed between January 2012 and October 2019 at another site (the Southwest Hospital) as an external testing dataset. The dataset comprised 87 patients with anti-NMDAR encephalitis (52 women; mean age: 44.24 ± 15.10 years). These patients were equally randomly divided into a training group (n = 61) and an internal testing group (n = 26) in a 7:3 ratio. The studies involving human participants were reviewed and approved by the Institutional Review Board of the First Affiliated Hospital of Chongqing Medical University (approval number 2016-67). The patients provided their written informed consent to participate in this study.

**Table 1:** Clinical variables associated with poor functional outcomes at the 24-month follow-up in 139 patients with adult anti-NMDAR encephalitis.

Variables	No. of patients (%)		Univariate analysis	
	Good outcome (n = 105)	Poor outcome (n = 34)	OR (95%CI)	*P-v*alue
**Age, mean (SD), y**	31.17 (± 14.81)	40.68 (± 14.15)	0.968 (0.939-0.996)	0.029^*^
**Gender (female)**	64 (61.0)	17 (50.0)	0.652 (0.263-1.629)	0.355
**Symptoms**				
Abnormal psychiatric/behaviour	65 (61.9)	25 (73.5)	0.320 (0.107-0.849)	0.029^*^
Seizures	61 (56.5)	17 (50.0)	1.354 (0.544-3.336)	0.510
Dyskinesias and movement disorders	15 (14.3)	14 (41.2)	0.159 (0.055-0.437)	<0.001^*^
Cognitive dysfunction	28 (26.7)	18 (52.9)	0.346 (0.136-0.866)	0.024^*^
Decreased consciousness	23 (21.9)	26 (76.5)	0.106 (0.035-0.289)	<0.001^*^
Autonomic instability	25 (23.8)	6 (17.6)	1.523 (0.530-5.069)	0.457
Speech disorder	30 (28.6)	22 (64.7)	0.214 (0.080-0.541)	0.001^*^
**Prodromal symptoms**	36 (34.3)	14 (41.2)	2.782 (1.048-8.342)	0.049^*^
**Complications**				
Pneumonia	36 (34.3)	14 (41.2)	0.979 (0.387-2.591)	0.964
Hypohepatia	25 (23.8)	9 (26.5)	0.798 (0.276-2.517)	0.685
Electrolyte disturbance	10 (9.5)	6 (17.6)	0.489 (0.141-1.798)	0.260
Urinary tract infections	22 (21.0)	8 (23.5)	0.704 (0.263-1.973)	0.490
Gastrointestinal bleeding	17 (16.2)	2 (5.9)	4.847 (0.873-90.894)	0.140
**ICU admission**	58 (55.2)	29 (85.3)	0.207 (0.056-0.605)	0.008^*^
**Tracheotomy**	4 (3.8)	7 (20.6)	0.212 (0.050-0.811)	0.025^*^
**Hospitalization days, mean (SD), d**	26.74 (± 17.70)	33.95 (± 32.10)	1.008 (0.993-1.032)	0.420
**Relapse**	16 (15.2)	18 (52.9)	0.133 (0.047-0.039)	<0.001^*^
**Rescue**	69 (65.7)	27 (79.4)	0.464 (0.141-1.305)	0.169
**Status epilepticus**	11 (10.5)	7 (20.6)	0.951 (0.418-2.624)	0.907
**Physical exam**				
Meningeal irritation sign	19 (18.1)	8 (23.5)	0.875 (0.306-2.740)	0.809
Pyramid sign	20 (19.0)	20 (70.6)	0.200 (0.074-0.507)	0.001^*^
**Time to start of treatment after symptom onset**			0.934 (0.895-0.966)	<0.001^*^
**Tumor**	8 (7.6)	4 (11.8)	0.348 (0.077-1.580)	0.158
**Initial mRS, mean (SD)**	4.35 (± 0.69)	3.85 (± 0.10)	0.529 (0.290-0.894)	0.026^*^
**Treatments**				
No use of immunotherapy	8 (7.6)	11 (32.4)	0.234 (0.067,0.785)	0.018^*^
First-line immunotherapy	44 (41.9)	21 (61.8)	0.389 (0.137-1.010)	0.061
Adding second-line immunotherapy	34 (32.4)	1 (2.9)	22.887 (4.645-414.634)	0.625
**CSF results**				
Weakly positive CSF antibody titers	50 (47.6)	18 (52.9)	0.929 (0.379-2.265)	0.870
Positive CSF antibody titers	35 (33.3)	5 (14.7)	1.760 (0.619-5.822)	0.314
Strongly positive CSF antibody titers	25 (23.8)	11 (32.4)	0.567 (0.235-1.326)	0.186
CSF pleocytosis	72 (68.6)	22 (64.7)	1.127 (0.437-2.816)	0.780
CSF abnormal protein	38 (36.2)	13 (38.2)	0.860 (0.348-2.168)	0.744
CSF abnormal glucose	15 (14.3)	7 (20.6)	0.910 (0.299-3.130)	0.873
CSF abnormal chloride	6 (5.7)	1 (2.9)	1.576 (0.220-31.602)	0.690
**Blood results**				
Weakly positive serum antibody titers	15 (14.3)	7 (20.6)	0.591 (0.207-1.777)	0.332
Positive serum antibody titers	20 (19.0)	3 (8.8)	3.125 (0.793-20.852)	0.151
Strongly positive serum antibody titers	4 (3.8)	3 (8.8)	0.970 (0.237-6.076)	0.969
Elevated leucocyte	53 (50.5)	15 (44.1)	1.181 (0.485-2.921)	0.715
Elevated neutrophil	61 (58.1)	15 (44.1)	1.767 (0.724-4.396)	0.213
**Abnormal ECG**	22 (21.0)	11 (32.4)	0.762 (0.287-2.125)	0.590
**Abnormal EEG**	82 (78.1)	24 (70.6)	1.714 (0.528-5.217)	0.349
**Extreme delta brush**	29 (27.6)	12 (35.3)	0.785 (0.319-1.950)	0.597
**Abnormal conventional MRI**	61 (58.1)	27 (79.4)	0.464 (0.141-1.305)	0.169

*P < 0.05.

Anti-NMDAR, anti-N-methyl-D-aspartate receptor; SD, standard deviation; OR, odds ratio; ICU, intensive care unit; mRS, modified Rankin Scale; CSF, cerebrospinal fluid; ECG, electrocardiogram; EEG, electroencephalogram.

Reference interval: CSF WBC count: 0–5 × 106/L; CSF protein level, 200 – 400 mg/L; CSF glucose level, 2.5 – 4.4 mmol/L; CSF chloride level, 120 – 130 mmol/L; blood WBC count, 3.5–10 × 10^9^/L; blood neutrophil count, 1.8-6.3×10^9^/L; weakly positive CSF antibody titers, 1:1; positive CSF antibody titers, 1:3.2 - 1:10; strongly positive CSF antibody titers, ≧ 1:32; weakly positive serum antibody titers, 1:10; positive serum antibody titers, 1:32 - 1:100; strongly positive serum antibody titers, 1:320.

### Prognostic Evaluation and Operational Definitions

Two experienced neurologists objectively and independently evaluated follow-up information at 4, 8, 12, 18, and 24 months after symptom onset, based on Titulaer’s previous study (5). Clinical relapse of anti-NMDAR encephalitis was defined as new onset or worsening of symptoms after at least 2 months of improvement or stability. All patients underwent at least one systemic tumor screening with an ultrasound scan, enhanced computed tomography, and/or tumor markers. We excluded patients with a follow-up of less than 4 months from the prognostic assessment.

The modified Rankin Scale (mRS) was used to assess the prognosis of patients. Dichotomous outcome status at 24 months was used as the ground truth for clinical, radiomics and DL analyses. A good outcome was defined as an mRS score ≤ 2, which ranged from fully recovered (mRS = 0) to mildly disabled but able to take care of oneself independently (mRS = 2). In contrast, a poor outcome (defined as mRS > 2) represented the range from moderate disability (mRS = 3) assistance with activities of daily living to severe disability (mRS = 5) requiring continuous care and death (mRS = 6).

### MR Image Acquisition and Hippocampus Annotation

MRI scans were performed using two 3.0 T scanners (GE Healthcare, Milwaukee, WI; Siemens Healthcare, Erlangen, Germany) with an eight-channel head coil and a 20-channel head-neck coil respectively in our hospital. Three 2D axial fast spin-echo sequences, including T_1_-weighted imaging (T_1_WI), T_2_-weighted imaging (T_2_WI) and fluid-attenuated inversion recovery imaging (FLAIR) sequences, and a 2D axial fast spin-echo echo-planar diffusion weighted imaging (SE-EP DWI) sequence were collected. The independent external data were collected using a 3.0 T scanner (Siemens Healthcare, Erlangen, Germany) at another site (Southwest Hospital). The more detailed parameter settings are displayed in **Table 2**.

**Table 2:** Parameters setting of the MRI scanning in our hospital.

Manufacturer	GE MEDICAL SYSTEMS				SIEMENS MEDICAL SYSTEMS			
Sequence	T_1_WI	T_2_WI	FLAIR	DWI	T_1_WI	T_2_WI	FLAIR	DWI
**TR (ms)**	2,050	4300	7600	4800	240	4030	9000	3300
**TE (ms)**	8.7	106	148	82	4.88	94	120	84
**Slice thickness (mm)**	5	5	5	5	5	5	5	5
**Spacing between slices (mm)**	6.5	6.5	6.5	6.5	6.5	6.5	6.5	6.5
**FOV (cm^2^)**	2.4×2.4	2.4×2.4	2.4×2.4	2.4×2.4	2.0×2.4	2.0×2.4	2.0×2.6	2.6×2.6
**Matrix size**	320×192	288×224	288×192	128×130	256×156	256×156	256×184	128×128
**Flip angle**	90	90	90	90	90	90	90	90
**NEX**	1	1	1	1	1	2	1	3

TR, repetition time, TE, echo time, FOV, field of view, NEX, number of excitation.

All of the patients’ multiparametric MRI data were uploaded to a commercial research platform (inferScholar, infervision, Beijing, China; http://research.infervision.com) for deidentification and annotation of 3D images. The region of interest (ROI) delineation method was the same as in one of our previous studies (21). Two neuroradiologists with 5 and 20 years of experience who were blinded to the clinical information outlined representative bilateral hippocampal areas on axial images from the four MRI sequences (T_1_WI/T_2_WI/FLAIR/DWI). The inter- and intraobserver reproducibility of ROI delineation was assessed using intra- and interclass correlation coefficients (ICCs). We initially chose 40 random images for independent ROI segmentation by two neuroradiologists. Within a 1-week period, each reader repeated the same manual procedure a second time to evaluate intraobserver reproducibility. Good agreement was defined as an ICC greater than 0.75.

### Data Preprocessing

Whole-brain MR images (T_1_WI, T_2_WI, FLAIR and DWI) as Digital Imaging and Communications in Medicine (DICOM) files from the Picture Archiving and Communication System (PACS) were exported for data preprocessing. All images were converted to Portable Network Graphics (PNG) format without annotation using the Python programming language (version 3.8.3) and the Nibabel library (version 3.2.1), scaled to 171 × 128 pixels, and randomly flipped in both directions. Next, we transformed each series of PNG images into an audio video interactive (AVI) video, reduced the video’s pixels per frame to 112 × 112, and then randomly extracted 16 frames into the 3D convolutional neural network.

Additionally, all whole-brain images were matched in space location, orientation, and origin to annotate the bilateral hippocampal images one by one for further radiomics analysis. Isotropic 3D resampling of DICOM images was performed by adjusting the X, Y and Z spacing size to 1 × 1 × 1 mm with linear interpolation. The signals were then smoothed with a Gaussian filter with a standard deviation of 0.5. To compensate for inhomogeneity artifacts and a lack of template intensity distribution, bias field correction and intensity standardization (gray level discretization from 0 to 255) were also applied.

### Clinical Model Building

The clinical model was constructed using univariate and multivariate logistic regression methods. Clinical characteristics were screened using univariate analysis to find independent predictors of poor prognosis. Variables with a *P-*value less than 0.05 were considered statistically significant. Then, for subsequent modeling, significant clinical variables were included, and a clinical model was developed using multivariate logistic analysis. We used bootstrap sampling to draw the calibration curve, and the data were sampled 1000 times. ROC curves were used to describe the predictive ability of the model. The relationships between various variables in the predictive model were described using a nomogram.

### DL-Based Predictive Model Building

Since 3D convolutional neural networks are computationally expensive, the R(2 + 1)D network separates the original spatio-temporal 3D convolution into a 2D spatial convolution and a 1D temporal convolution (22). A previous study found that R(2 + 1)D network was superior to other 3D convolutional neural networks in recognition tasks while keeping network parameters similar to those of other 3D backbone networks (23). As a result, we chose the R(2 + 1)D network, which is a relatively new image classification and segmentation architecture.

**Figure 1:**
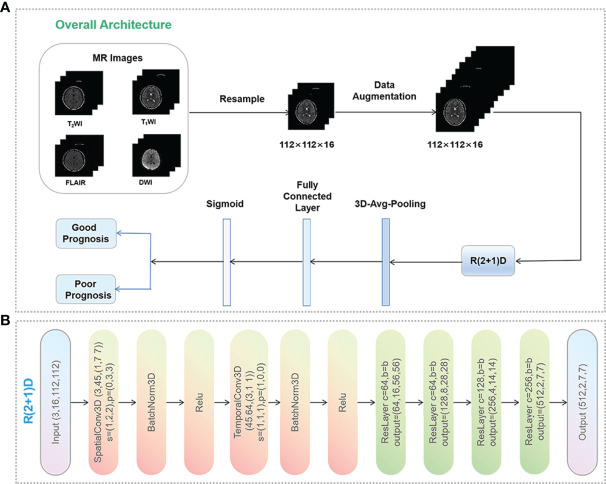
Data preprocessing pipeline. **(A)** Data preprocessing process and the workflow of the deep learning model. Data augmentation was performed only in the training set, including random reduction, center reduction, random flipping and brightness adjustment. **(B)** Overall architecture of R(2 + 1)D network. C, s, p, and b represent the number of input channels, the step size of the 3D convolution kernel, the size of padding, and spatio-temporal Resblock module, respectively. This module is a residual network structure. In the convolution layers of layer 1, layer 3, layer 4 and layer 5 of the model, the spatio-temporal Resblock module performs down sampling. The input tensor is (C, x, y) and the output tensor is (out_channels, X/2, Y/2). In the second layer of the model, the spatio-temporal Resblock module is not downsampled, and the input and output tensor shapes are the same.

Five DL models trained on four single or combined MRI sequences (T_1_WI/T_2_WI/FLAIR/DWI) were developed (**Figure 1**). First, we compressed the whole-brain images from the four sequences into 112 × 112 × 16 formats and fed them into the R(2 + 1)D network. The network outputs were successively passed through the 3D average pool layer and the fully connected layer. It was converted to a class probability vector by a sigmoid activation function as the prediction result.

Traditional data augmentation techniques such as rotation, zooming, flipping, and cropping were applied to process the 3D patches to artificially increase the training images up to eight times in the training set. To ensure the validity of the prediction results, this study did not perform data augmentation in the validation and test sets. In the training process, we used the SGD optimization strategy, with an initial learning rate of 0.01. After 30 iterations, the learning rate was multiplied by 0.1, the momentum was 0.9, the weight decay was 0.0001, and the model was implemented on the PyTorch library with 4 NVIDIA GPUs (GeForce GTX 3060Ti).

### Radiomics-Based Predictive Model Building

We used the Pyradiomics (https://pyradiomics.readthedocs) open source toolkit to extract features from each slice of the MR images of the annotated bilateral hippocampal areas. Radiomics features were extracted from each of the T_1_WI, T_2_WI, FLAIR and DWI images, which comprise first-order statistical features, shape- and intensity-based features, and high-order textural features such as gray-level cooccurrence matrix (GLCM), gray-level run-length matrix (GLRLM), gray-level size zone matrix (GLSZM), gray-level dependence matrix (GLDM) and neighborhood gray-tone difference matrix (NGTDM) (24). Finally, a total of 4420 features were extracted.

**Figure 2:**
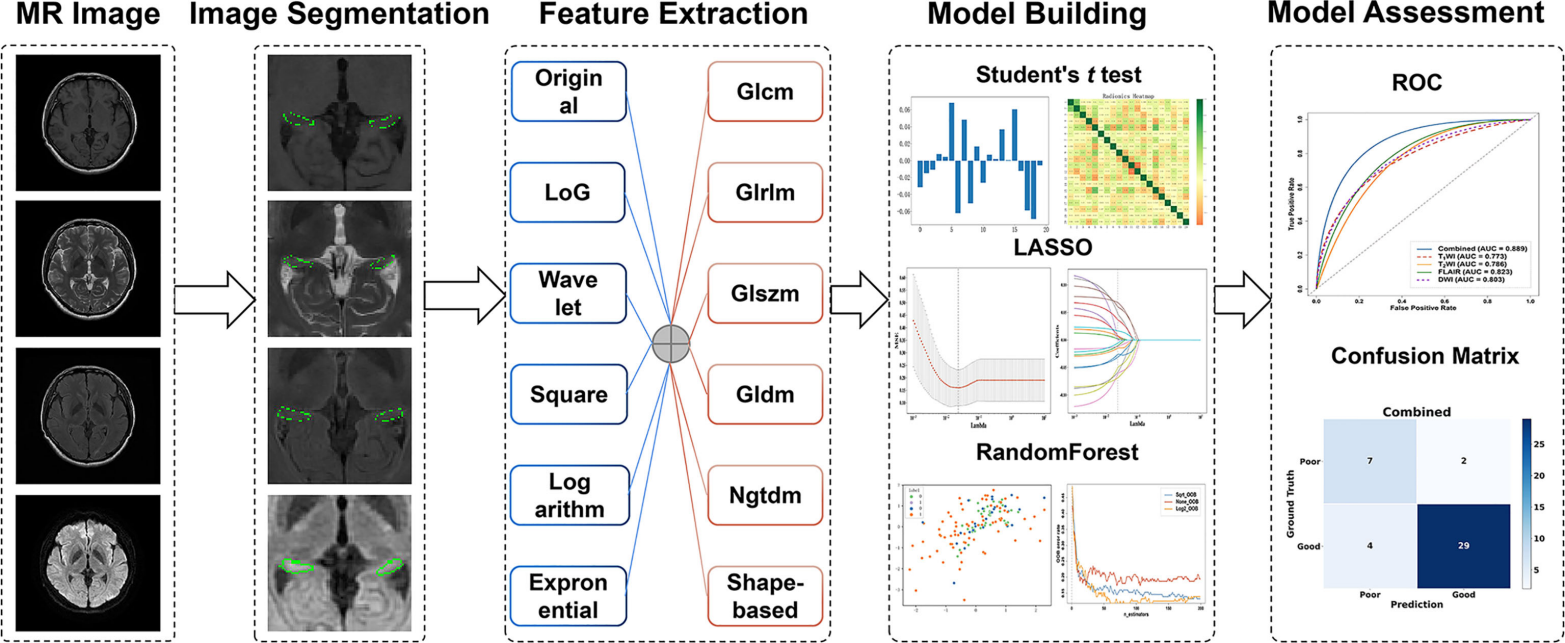
Radiomics workflow in the study.

In order to improve the generalization of features and optimize the model, Student’s *t*-test, least absolute shrinkage and selection operator (LASSO), and principal component analysis (PCA) were used to select radiomics features. Finally, the optimal method for feature selection was determined to be the Student’s *t*-test followed by the LASSO regression model. Then, the random forest algorithm was utilized to construct the predictive model. We used an out-of-bag error curve to evaluate the performance of the model and determined the number of subtrees to be 130 (25). **Figure 2** shows the detailed radiomics workflow.

### Fusion Model

A stacking algorithm, a subset of ensemble learning, combines the clinical, DL, and radiomics models to develop a new machine learning framework. The stacking algorithm refers to training one model to integrate data from multiple models (26). The clinical, DL_combined, and radiomics _combined models were built separately, and their prediction results were then input to the fusion model. These inputs were fed into a multivariate logistic regression model to obtain the final output as the predictions of the fusion model. We chose two layers for the fusion model since more layers increase the probability of overfitting.

### Model Evaluation

Due to our relatively small amount of data, all four models (clinical, DL, radiomics and fusion) were evaluated based on fivefold cross-validation. We randomly divided the samples into five subsets, with four subsets as the training set and one subset as the test set. To further reduce overfitting, a 12% no-replacement sampling of the training set was performed and the sampling results were put into the test set. The above operation was repeated in each folded cross-validation, and the sample size of the test set was 30% of the total sample size based on fivefold cross-validation. We applied this method to all four models.

The area under the receiver operating characteristic curve (AUC) and accuracy were used to evaluate the performance of different models. The Delong test was applied to test for significant differences in the ROCs between the fusion model and the clinical model, DL models and radiomics models in the internal and external datasets (*P-*value <0.05 was considered significant). To validate the generalizability of the nomograms, stratified analyses were performed using the Delong test on the subgroups of age, gender and MRI versions.

### Statistical Analysis

Statistical analysis was performed using SPSS (version 24.0; IBM, New York, USA) and R software (version 4.1.1; http://www.Rproject.org). The *t*–tests or Mann–Whitney *U*-tests were performed to compare continuous variables, while chi-squared or Fisher’s exact tests were used for classifying variables between groups. The Delong test was used to assess the difference in ROCs between clinical, DL, radiomics, and fusion models. The paired Student *t*-test was applied to compare the predictive performance of the fusion DL model to other clinical, DL, and radiomics models. A *P-*value < 0.05 was considered statistically significant.

## Results

### Clinical Characteristics

Of a total of 185 patients, 139 had complete clinical data with functional status at 24 months and were included for the univariate analysis. Of these 139 patients, 105 (75.5%) had a good functional outcome at 2 years, while 34 (24.5%) had a poor functional outcome. The associations between epidemiological, clinical, laboratory data, and treatment information and functional outcomes at 24 months are summarized in **Table 1**. Univariate analysis revealed that poor outcomes were associated with clinical data such as age (*P* = 0.029), symptoms (abnormal psychiatric/behaviour, *P* = 0.029; dyskinesias and movement disorders, *P* < 0.001; cognitive dysfunction, *P* = 0.024; decreased consciousness, *P* < 0.001; speech disorder, *P* = 0.001, prodromal symptoms, *P* = 0.049), and no use of immunotherapy (*P* = 0.018). In addition, ICU admission (*P* = 0.008), tracheotomy (*P* = 0.025), relapse (*P* < 0.001), pyramid sign (*P* = 0.001), time to start of treatment after symptom onset (*P* < 0.001), and initial mRS (*P* = 0.026) were associated with worse prognosis of anti-NMDAR encephalitis. In contrast, there were no significant differences in laboratory results, including CSF results, blood results, ECG, EEG and conventional MRI results (*P >* 0.05) (**Table 1**).

We found that dyskinesias and movement disorders, decreased consciousness, relapse and time to start of treatment after symptom onset were the most important factors for predicting poor functional outcomes of anti-NMDAR encephalitis (*P* < 0.001), and were significantly better predictors than other clinical characteristics (**Table 1**).

### Predictive Performance of the Clinical, DL and Radiomics Models

**Figure 3:**
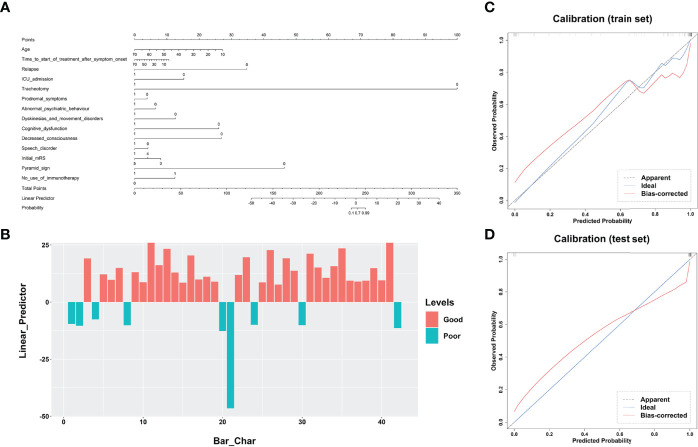
Performance of the clinical model’s nomogram. **(A)** The clinical variable-based nomogram revealed the significant factors for predicting poor outcomes of anti-NMDAR encephalitis. **(B)** The final total points are calculated by summing the score of each point represented for each variable. The prediction score of each patient is shown in the test dataset. Calibration curves of the clinical model are displayed in the training set **(C)** and validation set **(D)**. The predicted probabilities are shown on the x axis and the actual observed probability is represented on the y axis. The closer the two are to the dotted line, the better the prediction outcome. anti-NMDAR, anti-N-methyl-D-aspartate receptor.

All clinical variables with a *P-*value < 0.05 in **Table 1** were included in a multivariate logistic regression model. As shown in **Table 3** and **Figure 3**, the clinical model achieved high performance with an AUC of 0.840 (95% CI: [0.774-0.973]) and a consistently high accuracy of 0.905. The clinical variable-based nomogram was built to reveal the significant factors for predicting poor outcomes of anti-NMDAR encephalitis (see **Figure 3A**). The nomogram calibration curve of the clinical model demonstrated good agreement between prediction and observation in both the training and testing datasets (see **Figures 3C, D**).

**Table 3:** Performance measurements generated by clinical model, DL models and radiomics models trained on different sequences in the internal test dataset.

Models	AUC (95% CI)	*P-*value	Accuracy	Specificity	Sensitivity
**Clinical Variables**	0.840 (0.774-0.973)	0.047*	0.905	0.914	0.857
**Radiomics_T_1_WI**	0.773 (0.686-0.930)	0.036*	0.833	0.882	0.625
**Radiomics_T_2_WI**	0.786 (0.686-0.930)	0.038*	0.833	0.906	0.600
**Radiomics_FLAIR**	0.823 (0.632-0.897)	0.004*	0.786	0.929	0.500
**Radiomics_DWI**	0.803 (0.686-0.930)	0.014*	0.833	0.906	0.600
**Radiomics_Combined**	0.889 (0.715-0.946)	0.029*	0.857	0.936	0.636
**DL_T_1_WI**	0.721 (0.659-0.914)	0.035*	0.810	0.838	0.600
**DL_T_2_WI**	0.747 (0.560-0.861)	0.032*	0.738	0.806	0.333
**DL_FLAIR**	0.771 (0.659-0.914)	0.014*	0.810	0.903	0.546
**DL_DWI**	0.805 (0.686-0.930)	0.011*	0.833	0.861	0.667
**DL_Combined**	0.845 (0.715-0.946)	0.019*	0.857	0.886	0.714
**Fusion**	0.963 (0.874-0.999)	Na	0.976	1.000	0.900

*P < 0.05. The paired Student t-test was used to compare the prediction performance of the prognosis in patients with anti-NMDAR encephalitis between the fusion model and all the other models (The fusion model is the reference).

AUC, area under the receiver operating characteristic curve; CI, confidence interval; DL, deep learning; anti-NMDAR, anti-N-methyl-D-aspartate receptor; Na, Not available.

**Figure 4:**
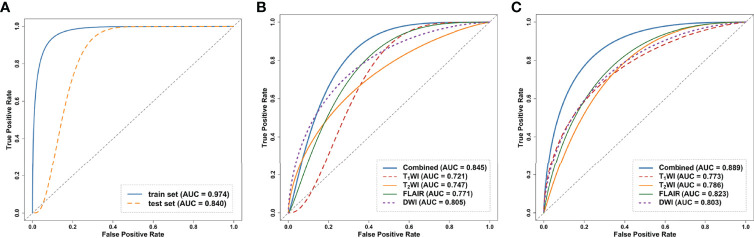
Receiver operating curves (ROC) of the models on the internal test dataset. **(A)** ROC of clinical model on the train and test dataset. **(B)** ROC curves of DL models trained on the four single sequences (T_1_WI/T_2_WI/FLAIR/DWI) and combined sequence. **(C)** ROC curves of the radiomics models trained on the four single sequences and combined sequence. ROC, receiver operating characteristic curve; DL, deep learning.

Regarding the DL models, the ROC curves (**Figure 4**) showed that DL models using T_1_WI, T_2_WI and FLAIR sequences had AUCs of 0.721 (95% CI: [0.659-0.914]), 0.747 (95% CI: [0.560-0.861]) and 0.771 (95% CI: [0.659-0.914]), respectively, which were lower than that of a model using the DWI sequence with an AUC of 0.805 (95% CI: [0.686-0.930]). The DL_combined model that was trained with the four MRI sequences combined exhibited higher performance than that of any single-sequence models, with an AUC of 0.845 (95% CI: [0.715-0.946]) and an accuracy of 0.857.

The intraobserver ICCs ranged from 0.865 to 0.998 and the interobserver ICCs ranged from 0.792 to 0.958, indicating satisfactory inter- and intraobserver reproducibility of manual delineation. All radiomics models using a single sequence showed comparable performance with AUCs of 0.773 (95% CI: [0.686-0.930]) for T_1_WI, 0.786 (95% CI: [0.686-0.930]) for T_2_WI, 0.823 (95% CI: [0.632-0.897]) for FLAIR and 0.803 (95% CI: [0.686-0.930]) for DWI. The radiomics_combined models provided greater performance than any of the single-sequence models, with an AUC of 0.889 (95% CI: [0.715-0.946]) and a desirable accuracy of 0.857 (**Table 3** and **Figure 4**).

**Figure 5:**
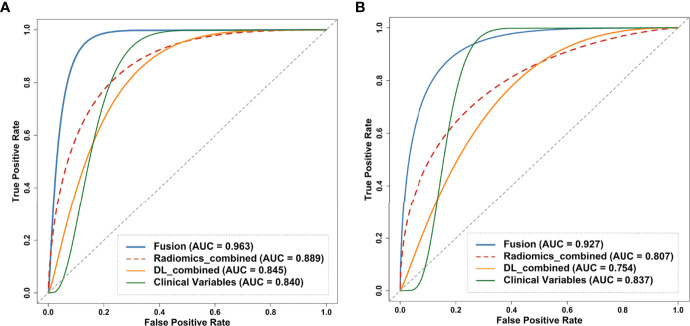
Receiver operating curves (ROC) of the clinical model, DL_combined model, radiomics_model and fusion model on the **(A)** internal and **(B)** external test dataset. Fusion model was developed by combing clinical variables, DL_combined features and radiomics_combined features. ROC, receiver operating characteristic curve; DL, deep learning.

**Figure 6:**
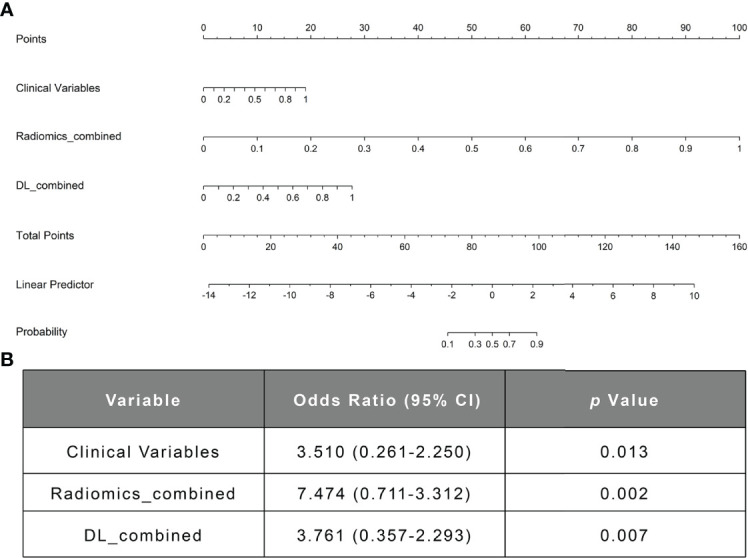
The nomogram of the fusion model. **(A)** The nomogram of the fusion model combining the clinical model’s prediction score, the DL-based image prediction score, and the radiomics-based image prediction score. **(B)** The multivariable logistic regression analysis of the clinical variables, the radiomics_combined model, and the DL_combined model. DL, deep learning.

In the comparison across models, the DL model trained with combined sequences and the radiomics model trained with combined sequences had higher AUCs and accuracies than the single-sequence models, and the predictive performance of the radiomics_combined model was superior to that of the DL_combined model and clinical model (**Table 3** and **Figure 5A**). The fusion model integrated by the predictor scores based on clinical, DL_combined, and radiomics_combined models performed significantly better than all other models, with an AUC of 0.963 [95% CI: (0.874-0.999)] and a satisfactory accuracy of 0.976 in the internal dataset (*P* < 0.05). As shown in **Table 4** and **Figure 5B**, the fusion model consistently significantly outperformed all other models, with an AUC of 0.927 (95% CI: [0.688-0.975]) and an accuracy of 0.880 in the independent external dataset (*P* < 0.05). The nomogram of the fusion model was built to help predict the prognosis of anti-NMDAR encephalitis (**Figure 6A**). All three variables (clinical variables, DL-based imaging predictors, and radiomics-based imaging predictors) were clinically and significantly predictive of functional outcomes in anti-NMDAR encephalitis (**Figure 6B**). **Supplementary Figure 2** shows the confusion matrix for the internal testing dataset of all the models.

**Table 4:** Performance measurements generated by DL models and radiomics models trained on four combined sequences (T_1_WI/T_2_WI/FLAIR/DWI) and clinical model trained on clinical variables in the external test dataset.

Models	AUC (95% CI)	*P-*value	Accuracy	Specificity	Sensitivity
**Clinical Variables**	0.837 (0.639-0.955)	0.017*	0.840	0.818	1.000
**Radiomics_Combined**	0.807 (0.549-0.906)	0.024*	0.760	0.889	0.429
**DL_Combined**	0.754 (0.639-0.955)	0.007*	0.840	0.900	0.600
**Fusion**	0.927 (0.688-0.975)	Na	0.880	0.947	0.667

*P < 0.05. The paired Student t-test was used to compare the predictive performance of the prognosis in patients with anti-NMDAR encephalitis between the fusion model and the other three models (The fusion model is the reference).

AUC, area under the receiver operating characteristic curve; CI, confidence interval; DL, deep learning; anti-NMDAR, anti-N-methyl-D-aspartate receptor; Na, Not available.

### Stability and Predictive Values of Models

As shown in **Supplementary Figure 3**, the stratified analyses showed that the predictive performances of the clinical, DL and radiomics nomograms were not influenced by patient age, gender, or MRI versions (Delong test: all *P* > 0.05). The details are presented in the **Supplementary Material**.

## Discussion

In this study, we constructed a fusion nomogram that combined DL- and radiomics-based imaging predictors from multiparametric MRI and a large of clinical variables to predict the functional outcomes of anti-NMDAR encephalitis early and effectively. The proposed fusion model achieved high predictive accuracy and significantly outperformed all other single-method-based models. The radiomics_combined model exceeded both the DL_combined and the clinical models, providing a better way to predict the disease outcomes. We developed an automated, pretreatment and individualized tool for the prognostic prediction of anti-NMDAR encephalitis, which could aid in the development of novel treatment strategies and improvement of patient prognosis.

Among the clinical risk factors, we found that dyskinesias and movement disorders, decreased consciousness and time to start of treatment after symptom onset were the most important univariate predictors, which was consistent with prior studies. In a retrospective study of 382 patients with anti-NMDAR encephalitis, Balu R et al. discovered that ICU admission, treatment delay, and movement disorder were the most important univariate predictors (9). A previous systematic study also found that decreased consciousness, ICU admission, and lack of immunotherapy were all related to poorer outcomes in anti-NMDAR encephalitis (7). In our study, several other clinical features such as older age, tracheotomy, pyramid sign, and symptoms (e.g., abnormal psychiatric/behaviour, dyskinesias and movement disorders, cognitive dysfunction, decreased consciousness, and speech disorder), were also found to be associated with poor prognosis in anti-NMDAR encephalitis. All of these risk factors are linked to severe anti-NMDAR encephalitis (27), suggesting that physicians should pay special attention to older anti-NMDAR patients and intervene early to avoid their conversion from mild to refractory severe encephalitis. A few studies have revealed that second-line immunotherapy could reduce the risk of recurrence, but the relationship between relapse and patient prognosis has not been well investigated (5, 9). Based on our findings, relapsed patients with anti-NMDAR encephalitis had a worse prognosis. Therefore, we emphasize that second-line immunotherapy should be given as soon as possible in the acute phase of the disease to reduce relapses and further improve prognosis.

In our previous study, DL methods using convolutional neural networks were used to effectively detect and characterize AE. However, the study did not utilize information from the whole brain but only from the bilateral hippocampal regions (21). Although the hippocampus is considered to be the characteristic structure involved in anti-NMDAR encephalitis, conventional MRI shows diffuse encephalitis across multiple brain regions, including the hippocampus (13, 28). In this study, we selected the whole-brain areas as signature ROIs and used them as inputs into the R(2 + 1)D network for prognostic analysis. Our findings showed satisfactory performance of DL models trained with whole-brain MRI features for predicting the prognosis of anti-NMDAR encephalitis, which could be useful for helping patients develop personalized treatment plans early.

Radiomics techniques have been widely applied to generate identification and prognostic biomarkers for neuropsychiatric diseases because they can assess and quantify a vast variety of imaging parameters to extract highly predictive imaging features (29–31). To our knowledge, the utilization of radiomics features based on multiparametric MRI to predict the prognosis of anti-NMDAR encephalitis has rarely been reported. Previous studies have shown that MRI findings of anti-NMDAR encephalitis, particularly in the hippocampal region, can help reveal the clinical features and disease outcomes (12, 28, 32). Finke et al. used advanced MRI to show that hippocampal atrophy and impaired microstructural integrity were associated with disease severity in patients with anti-NMDAR (33, 34). The existence of disease-specific damage in the hippocampal area was revealed by Heine et al., which was related to prognosis (12). We therefore chose to extract features from bilateral hippocampal regions for the radiomics prediction task. The results of the proposed radiomics models suggested that the extracted bilateral hippocampal features can be used as an effective biomarker for early prognostic prediction in anti-NMDAR encephalitis.

Our results showed that the combined model trained with all MRI sequences has superior predictive performance than single-sequence models from both DL and radiomics approaches. This suggested that multiparametric MRI parameters based on machine learning can improve prediction abilities by better comprehending the characteristics of anti-NMDAR encephalitis than single sequences (35, 36). In predicting the prognosis of anti-NMDAR encephalitis, the combined radiomics model marginally exceeded the DL_combined and clinical models. This could be attributed to machine learning’s high performance in analyzing medical images. The properties of radiomics make it more suitable for relatively small sample size of data than DL (37, 38).

With a high AUC of 0.963 and a satisfying accuracy of 0.976, the fusion framework combining clinical, DL_combined, and radiomics_combined models performed significantly better than all other models (*P* < 0.05). This artificial intelligence scheme appears to be a promising model for anti-NMDAR encephalitis prognostic prediction with broad development prospects.

There are several limitations to this study. First, we did not include biomarkers associated with treatment response because they were not available in the dataset, but these data could further improve the model’s capacity to predict ultimate clinical outcomes. Second, the bilateral hippocampal areas used for radiomics analysis were manually segmented layer by layer by experienced radiologists, which was time-consuming. Automated detection and segmentation of the hippocampal region is desirable. Finally, we developed a prognostic model based on clinical and machine learning methods for the early prediction of anti-NMDAR encephalitis, which could be extended to other subtypes of AE in the future. We will include more data on anti-NMDAR encephalitis and other subtypes of AE in further trials to improve the accuracy and clinical value of our model.

In conclusion, we provided an integrated fusion nomogram using clinical variables and machine learning imaging predictors based on multiparametric MRI. Our two-center results suggest that the fusion model could be used as a noninvasive computer-aided diagnostic tool for early identification of patients who may require more active monitoring. It also identifies patients with a poor prognosis who may experience relapses after receiving definitive treatment. These individuals could benefit from early second-line immunotherapy.

## Data Availability Statement

The original contributions presented in the study are included in the article/**Supplementary Material**. Further inquiries can be directed to the corresponding author.

## Ethics Statement

The studies involving human participants were reviewed and approved by The Institutional Review Board of the First Affiliated Hospital of Chongqing Medical University. The patients/participants provided their written informed consent to participate in this study.

## Author Contributions

YL, SC, YX, and XD had full access to all the data in the study and take responsibility for the integrity of the data and accuracy of the data analysis. YX, XD, YL, and SC conceived and designed the study. All authors acquired, or interpreted the data. YX and XD drafted the manuscript. YL and SC critically revised the manuscript for intellectual content. YX, XH, and JF collected and evaluated the data. XD statistically analyzed the data. SC and YX verified the underlying data. YL, CZ, and SC obtained funding. JL, HL, CZ, SD, and JW provided administrative, technical, or material support. YH and QL supervised the study. All authors contributed to the article and approved the submitted version.

## Funding

This study was supported by the Medicine Scientific Key Research Project of Chongqing Municipal Health and Family Planning Commission of China (NO.2016ZDXM002), the Chongqing Basic Research and Frontier Exploration Project of Chongqing Science and Technology Commission (NO.cstc2018jcyjAX0584), the Medical Scientific Youth Project of Chongqing Municipal Health and Family Planning Commission of China (NO.2018QNXM004) and the Chongqing Natural Science Foundation (cstc2019jcyj-msxmX0130).

## Conflict of Interest

The authors declare that the research was conducted in the absence of any commercial or financial relationships that could be construed as a potential conflict of interest.

## Publisher’s Note

All claims expressed in this article are solely those of the authors and do not necessarily represent those of their affiliated organizations, or those of the publisher, the editors and the reviewers. Any product that may be evaluated in this article, or claim that may be made by its manufacturer, is not guaranteed or endorsed by the publisher.
