# Supplementary material for: Clinical Variables, Deep Learning and Radiomics Features Help Predict the Prognosis of Adult Anti-N-methyl-D-aspartate Receptor Encephalitis Early: A Two-Center Study in Southwest China
Source: Front Immunol. 2022 Jun 1;13:913703. doi: 10.3389/fimmu.2022.913703 (PMC9199424; doi:10.3389/fimmu.2022.913703)
Supplement: Supplementary file 1 [file DataSheet_1.docx]

Supplementary Material


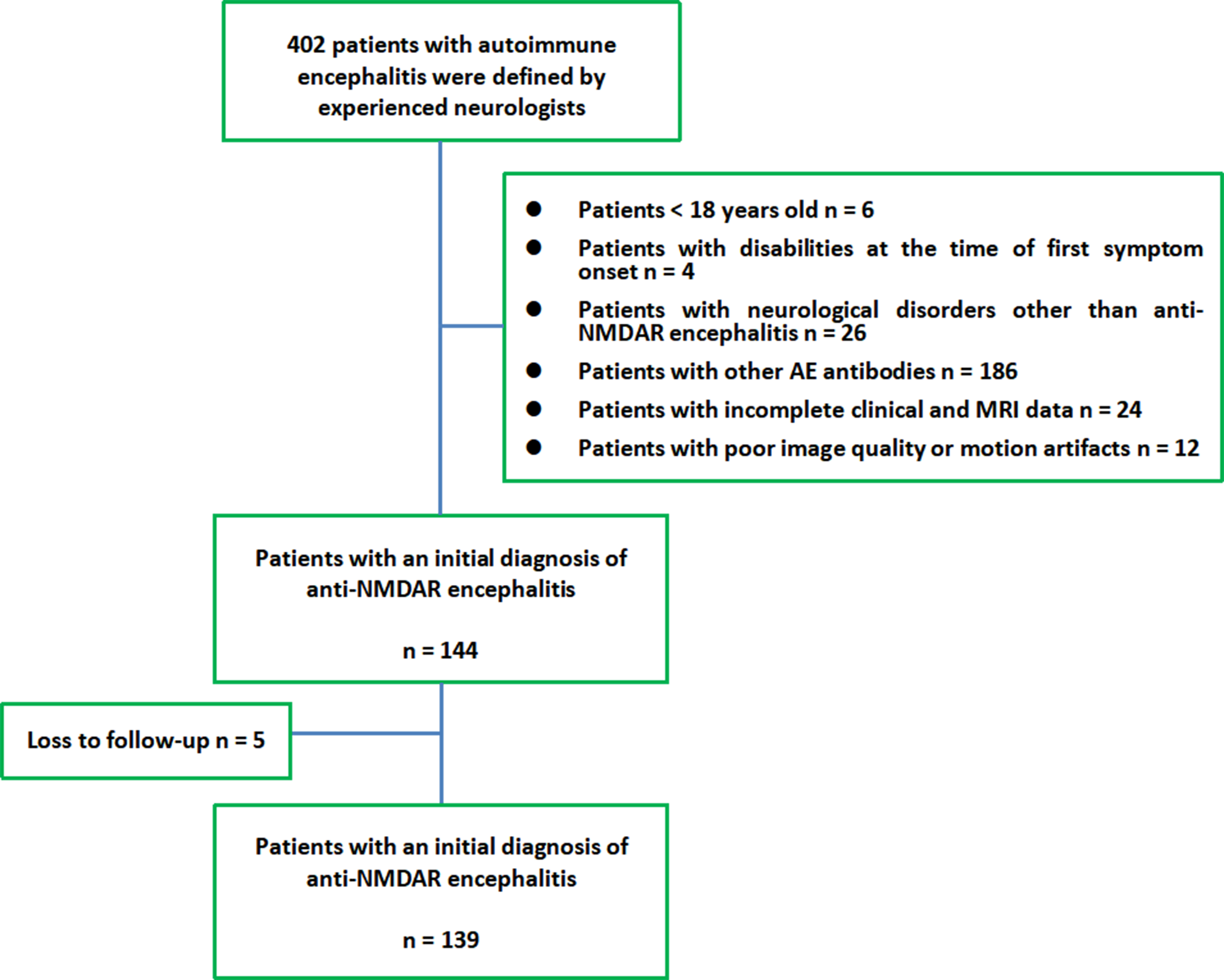


**Supplementary Figure 1.** Flowchart illustrated patients’ selection process. We prospectively collected clinical data and routine cranial MRI data from 402 anti-NMDAR patients for modeling and evaluation. Anti- NMDAR, anti-N-methyl-D-aspartate receptor.

**
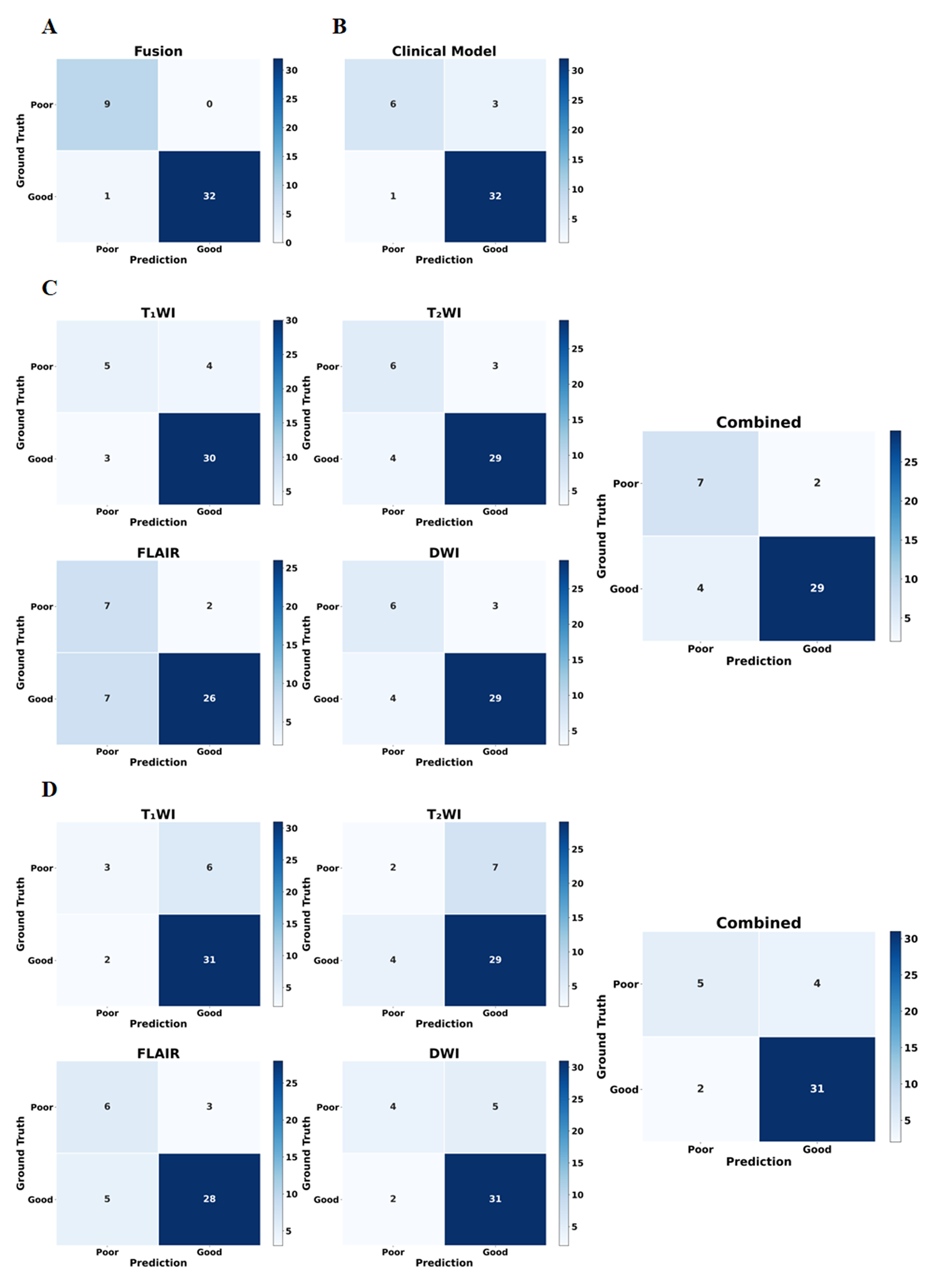
**

**Supplementary Figure 2.** Confusion Matrix for the internal testing dataset of the fusion model **(A)**, the clinical model **(B)**, radiomics models **(C)**, and DL models using four single or combined four MRI sequences **(D)**.

**Stratified analysis of fusion nomogram**

The generalizability of a nomogram is always of great concern in terms of its application values. In order to test the generalization ability of our fusion nomogram, we performed stratification analysis on the subgroups of sex, age, and version of MRI. We used the ROC curve and AUC to evaluate the performance of our nomogram on these subpopulations. The results showed that the fusion nomogram was not influenced by these factors (all *p* > 0.05), indicating a good generalizability of the nomogram.

**1. Stratified analysis on age：**Because the median age of the patients was 33, patients are divided into two subgroups: age <= 33 and age > 33 with AUC of 0.897 and 0.973 (Delong test *p* value >0.05 compared with the result on the overall cohort).

**2. Stratified analysis on gender：**Patients are divided into two subgroups: female and male with AUC of 0.971 and 0.942 (Delong test *p* value >0.05 compared with the result on the overall cohort).

**3. Stratified analysis on version of MRI system：**Patients are divided into two subgroups: scanned using GE MRI system and using SIEMENS MRI system with AUC of 0.977 and 0.899 (Delong test *p* value >0.05 compared with the result on the overall cohort).


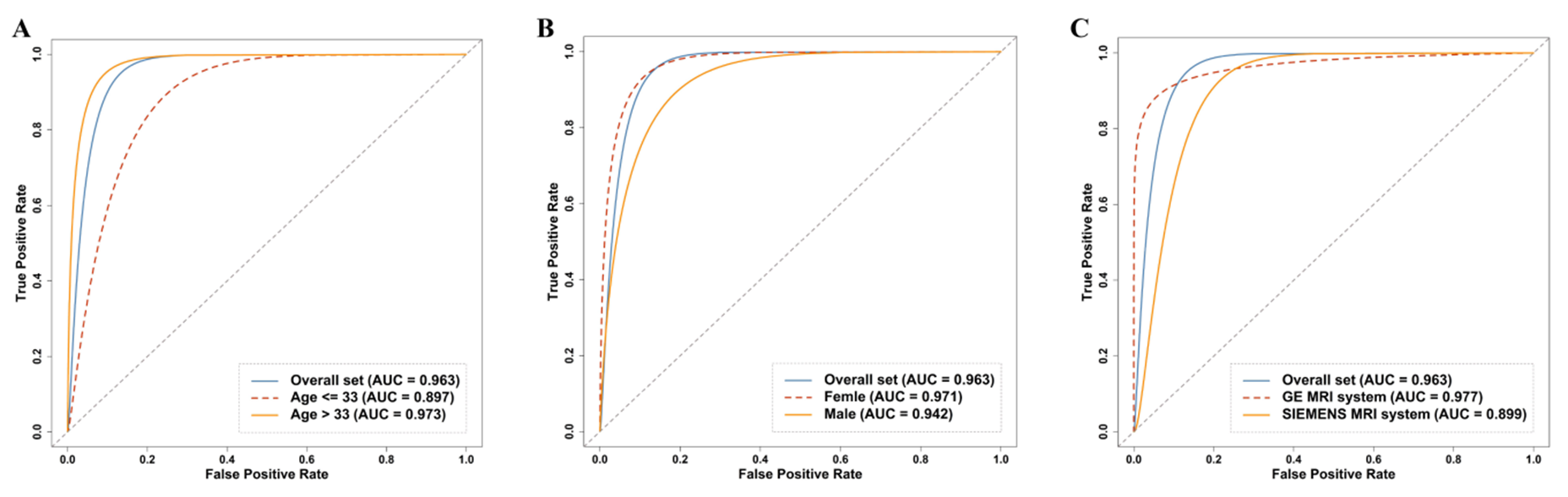
**Supplementary Figure 3.** The fusion nomogram score for each subgroup stratified by **(A)** age, **(B)** gender, and **(C)** version of MRI system.

**Sample size consideration**

To determine the minimum cases of patients, a power analysis was done before conducting the experiment. We used R package ‘pwr’ and two-sample paired *t* test to implement the test. We set the power to be 80% to find differences in sensitivity between two groups with significance level of 5%. With conservative estimate, the minimum sample size of 25 should be included in each group. We have met this requirement by assigning 42 patients in our internal test set (a total of 139 patients in dataset) and 26 patients in our external test set (a total of 87 patients in dataset).
